# Reconstruction of Long-Chain Polyunsaturated Acid Synthesis Pathways in Marine Red Microalga *Porphyridium cruentum* Using Lipidomics and Transcriptomics

**DOI:** 10.3390/md22020082

**Published:** 2024-02-09

**Authors:** Tao Li, Chulin Li, Weinan Wang, Hualian Wu, Houbo Wu, Jin Xu, Wenzhou Xiang

**Affiliations:** 1 CAS Key Laboratory of Tropical Marine Bio-Resources and Ecology, Guangdong Key Laboratory of Marine Materia Medica, Institution of South China Sea Ecology and Environmental Engineering, South China Sea Institute of Oceanology, Chinese Academy of Sciences, Guangzhou 510301, China; taoli@scsio.ac.cn (T.L.); lchlxpy@126.com (C.L.); wangweinan0220@163.com (W.W.); hlwu@scsio.ac.cn (H.W.); wuhoubo@scsio.ac.cn (H.W.); 2 Guangzhou Institute of Energy Conversion, Chinese Academy of Sciences, CAS Key Laboratory of Renewable Energy, Guangdong Provincial Key Laboratory of New and Renewable Energy Research and Development, Guangzhou 510640, China

**Keywords:** *Porphyridium*, EPA, lipidomics, ω3 and ω6 pathway, fatty acid desaturase

## Abstract

The marine red microalga *Porphyridium* can simultaneously synthesize long-chain polyunsaturated fatty acids, including eicosapentaenoic acid (C20:5, EPA) and arachidonic acid (C20:4, ARA). However, the distribution and synthesis pathways of EPA and ARA in *Porphyridium* are not clearly understood. In this study, *Porphyridium cruentum* CCALA 415 was cultured in nitrogen-replete and nitrogen-limited conditions. Fatty acid content determination, transcriptomic, and lipidomic analyses were used to investigate the synthesis of ARA and EPA. The results show that membrane lipids were the main components of lipids, while storage lipids were present in a small proportion in CCALA 415. Nitrogen limitation enhanced the synthesis of storage lipids and ω6 fatty acids while inhibiting the synthesis of membrane lipids and ω3 fatty acids. A total of 217 glycerolipid molecular species were identified, and the most abundant species included monogalactosyldiglyceride (C16:0/C20:5) (MGDG) and phosphatidylcholine (C16:0/C20:4) (PC). ARA was mainly distributed in PC, and EPA was mainly distributed in MGDG. Among all the fatty acid desaturases (FADs), the expressions of Δ5FAD, Δ6FAD, Δ9FAD, and Δ12FAD were up-regulated, whereas those of Δ15FAD and Δ17FAD were down-regulated. Based on these results, only a small proportion of EPA was synthesized through the ω3 pathway, while the majority of EPA was synthesized through the ω6 pathway. ARA synthesized in the ER was likely shuttled into the chloroplast by DAG and was converted into EPA by Δ17FAD.

## 1. Introduction

Long-chain polyunsaturated fatty acids (LC-PUFAs), mainly including eicosapentaenoic acid (C20:5, EPA), arachidonic acid (C20:4, ARA), and docosahexaenoic acid (C22:6, DHA), have been widely used in nutraceuticals, milk powder, and anti-acne products due to their functions in preventing cardiovascular diseases and promoting brain development, and their anti-inflammatory effects [1]. LC-PUFAs are very rare in higher plants and animals but are abundant in marine microalgae such as *Phaeodactylum tricornutum*, *Nannochloropsis oculata,* and *Porphyridium cruentum* [2]. The marine unicellular red alga *P*. *cruentum*, which belongs to Rhodophyta (phylum), Protoflorideophyceae (class), Porophyridiales (order), Porophyridiaceae (family), and *Porphyridium* (genus), can simultaneously synthesize high contents of ARA and EPA (>50% of total fatty acids). Thus, *P*. *cruentum* is considered an ideal experimental material for studying the synthesis pathways of LC-PUFAs [3,4].

In the synthesis of LC-PUFAs in microalgae, C18:1ω9 is firstly desaturated to C18:2ω6 by Δ12 fatty acid desaturase (Δ12FAD) [5]. Subsequently, C18:2ω6 is converted to EPA and ARA via the ω3 and ω6 fatty acid synthesis pathways, respectively [5]. The ω3 fatty acid synthesis pathway occurs in the following steps: C18:2ω6 → (Δ15FAD) → C18:3ω3 → (Δ6FAD) → C18:4ω3 → (elongase of very-long-chain fatty acid, ELOVL) → C20:4ω3 → (Δ5FAD) → EPA [5]. The ω6 fatty acid synthesis pathway is as follows: C18:2ω6 → (Δ6FAD) → C18:3ω6 → (ELOVL) → C20:3ω6 → (Δ5FAD) → ARA [5]. Among these fatty acid desaturases (FADs), Δ5FAD and Δ6FAD are located in the endoplasmic reticulum (ER) [6,7], while Δ12FAD and Δ15FAD are found in both the chloroplast and the ER. Δ5FAD, Δ6FAD, Δ12FAD, and Δ15FAD in the ER prefer to use phosphatidylcholine (PC) as a substrate [8]. Different intracellular compartments and substrate preferences of FADs create complex glycerolipid molecules with diverse types of fatty acid chains [9]. Storage lipids mainly include triacylglycerol (TAG), wax esters, and diacyl-alkylglycerols (DAGEs) in microalgae [8]. Membrane lipids are divided into eukaryotic and prokaryotic membrane lipids. Prokaryotic membrane lipids include monogalactosyldiaclyglycerol (MGDG), digalactosyldiaclyglycerol (DGDG), and phosphatidylglycerol (PG), all of which are synthesized only in the chloroplast and localized in the thylakoid membrane. Eukaryotic membrane lipids comprise PC and phosphatidylethanolamine (PE), which are synthesized in the ER [8]. EPA in most microalgae is found mainly in MGDG, such as in *P*. *tricornutum* [10], *Nannochloropsis* sp. [11], and *Eustigmatos vischeri* JHsu-01 [12]. Interestingly, EPA is synthesized outside the chloroplast [13]; however, it is unclear how extra-chloroplast EPA penetrates into the chloroplast to participate in the synthesis of MGDG [14]. Studies on *N*. *oceanica* IMET1 have shown that diglyceride (DAG) may act as a shuttle molecule to carry EPA into the chloroplast [15]. In addition to the well-known EPA synthesis by the ω3 fatty acid synthesis pathway, the synthesis of EPA by a new ω6 fatty acid synthesis pathway has also been reported in some fungi [16]. In these pathways, Δ17FAD is the key enzyme catalyzing the desaturation of ARA to EPA.

The ω6 fatty acid synthesis pathway of *Porphyridium* was investigated using stable isotope labeling technology and supplementation of intermediate fatty acids by many scientists in the late 1990s [3]. However, due to the limitations of technology at the time, many problems in this pathway have not been well resolved: (1) it is difficult to construct the whole EPA synthesis network based on a small number of glycerolipid molecules identified using thin-layer chromatography and gas chromatography [3]; (2) some key FADs are not clear (e.g., the intracellular localization and substrate preferences of Δ17FAD are unclear); and (3) the intermediate metabolites C18:4ω3 and C20:4ω3 in the ω3 synthesis pathway have not been detected, according to a large number of results from previous studies [17,18]. Therefore, the involvement of the ω3 and ω6 pathways in EPA synthesis remains controversial.

Lipidomics is a new branch of metabolomics that focuses on systematic studies of lipid metabolites and their interactions. At present, the largest lipid database (LIPID MAPS) comprises information from all over the world on the structures of more than 8000 lipid species, including free fatty acids, TAG, phospholipids, and glycolipids [19]. The fatty acid composition of glycerolipids from some microalgae has also been analyzed via lipidomics. For example, there are more than 20 TAG molecules with different fatty acid compositions in *Dunaliella* [20]. Han et al. identified a total of 41 glycolipid molecular species in *N*. *oceanica* IMET1 and reconstructed the synthesis pathway of EPA-containing chloroplast membrane lipids [15]. Diacylglyceryl-N,N,N-trimethylhomoserine (DGTS) is a central intermediate in glycerolipid remodeling, which replaces the carrier PC via C18 fatty acid desaturation. DGTS donates acyl groups (as precursors) to EPA, which is used in the formation of chloroplast glycolipids [21]. Thus, lipidomics has been demonstrated to be an ideal approach for studying algal lipid metabolism.

Microalgae accumulate storage lipids and decompose membrane lipids under nitrogen-limited conditions [12,15]. This makes it possible to study lipid metabolism by changing the nitrogen concentration. In this study, changes in the cellular ultrastructure and content of lipid fractions in *P*. *cruentum* CCALA 415 were investigated under nitrogen-replete and nitrogen-limited conditions. The distribution of EPA and ARA in different glycerolipid molecules was assessed based on lipidomics data. Changes in the expression levels of genes during glycerolipid synthesis were studied via transcriptomics. Finally, the synthesis networks of EPA and ARA in *P*. *cruentum* were revealed based on transcriptomics and lipidomics data. This study will provide guidance in respect of EPA and ARA synthesis and distribution in microalgae.

## 2. Results

### 2.1. Growth and Morphological Characteristics of P. cruentum CCALA 415 under N-Limited and N-Replete Conditions

**Figure 1:**
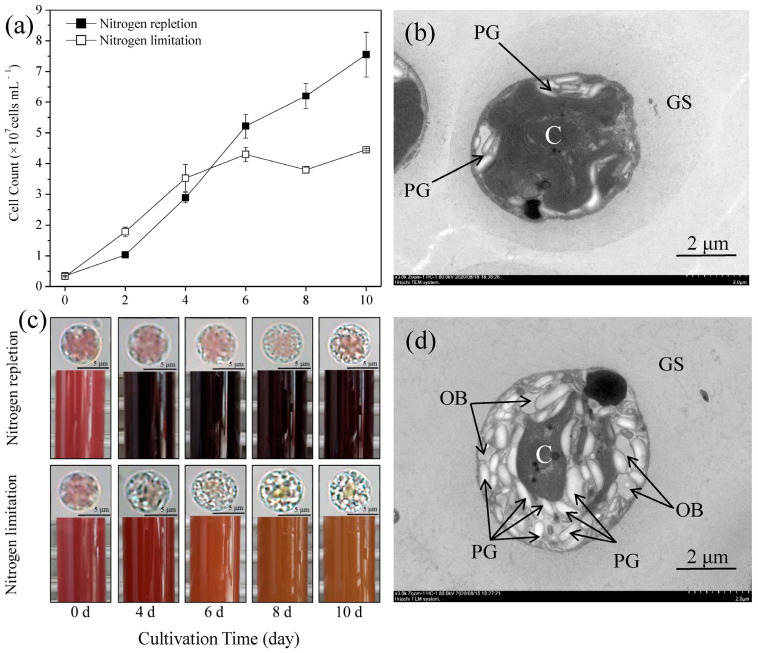
Growth characteristics of *Porphyridium cruentum* CCALA 415 under N-limited and N-replete conditions, expressed as (**a**) cell count; (**b**) culture and cellular morphology; (**c**) cell ultrastructure of N-replete groups on day 10; and (**d**) cell ultrastructure of N-limited groups on day 10. The values shown in Figure 1a are the averages of three biological replicates and three technical replicates ± standard deviation (*n* = 9). PG: polysaccharide granule; C: chloroplast; GS: gelatinous sheath; OB: oil body.

The cell count from day 0 to day 4 of the N-limited group was significantly higher than that of the N-replete group (*p* < 0.05). After 4 days, the cell count of the N-replete group exceeded that of the N-limited group. At the end of cultivation, the cell count of the N-limited group was 41.1% lower (4.45 × 10^7^ cells mL^−1^) than that of the N-replete group (7.55 × 10^7^ cells mL^−1^) (Figure 1a).

The N-limited and N-replete groups exhibited different culture colors (Figure 1c). With the prolonging of the culture time, the color of the N-limited group changed from purplish red to orange-yellow and the change in cell size (~5 μm) was not obviously observed. Additionally, the scattered purplish red chloroplasts gradually gathered in the centers of the cells. Transparent granules appeared in the cells, and their number gradually increased. In contrast, the color of the N-replete group remained purplish red. Transparent granules also appeared in the cells, but their number was less than that of the N-limited group.

The cellular ultrastructure was observed via transmission electron microscopy (Figure 1b,d). The extracellular gelatin sheath of cells cultured under the N-limited conditions was decomposed. The chloroplast had a smaller size and migrated to the center of the cell, and its lamellar structure disappeared. A large number of starch granules, along with a small number of oil bodies, were observed in the cells, indicating that the main storage form of carbon in *P*. *cruentum* is carbohydrates rather than storage lipids.

### 2.2. Lipid Accumulation of P. cruentum CCALA 415 Cultured under N-Limited and N-Replete Conditions

**Figure 2:**
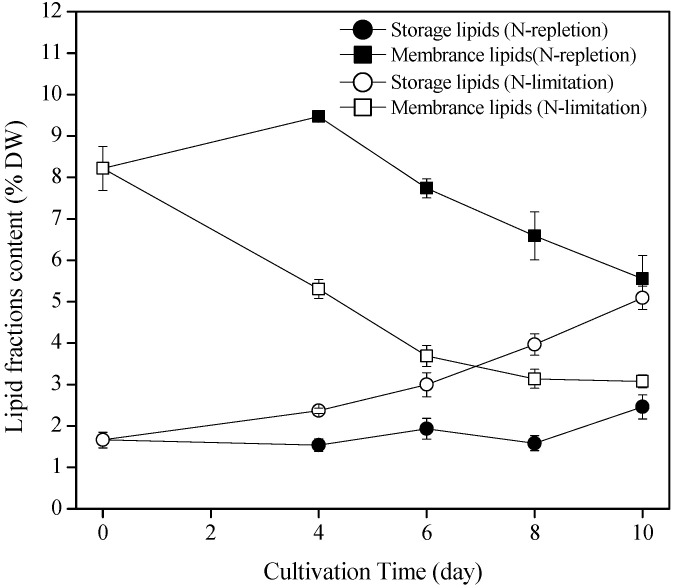
Lipid accumulation and fractionation of *Porphyridium cruentum* CCALA 415 under N-limited and N-replete conditions. The values shown are the averages of three biological replicates and three technical replicates ± standard deviation (*n* = 9).

The content of storage lipids in both the N-limited and N-replete groups gradually increased with the increase in culture time (Figure 2). The content of storage lipids in the N-limited group was significantly higher than that of the N-replete group at the same culture time. At the end of the culture (day 10), the storage lipids of the N-limited group increased to 5.10% DW from 1.66% DW, which was an increase of 1.07 times compared to that of the N-replete group (2.46% DW) (*p* < 0.05).

By contrast, the content of membrane lipids in both groups decreased with the increase in culture time. The maximum content of membrane lipids detected in the two groups was 9.47% DW, and this was observed on day 4. The membrane lipid content of the N-limited group was significantly lower than that of the N-replete group at the same culture time. At the end of the culture, the membrane lipid content of the N-limited and N-replete groups decreased to 3.08% DW and 5.56% DW, respectively, decreasing by 62.5% and 32.3%, respectively, compared to that on day 4 (*p* < 0.05).

### 2.3. Fatty Acid Content of P. cruentum CCALA 415 Cultured under N-Limited and N-Replete Conditions

**Figure 3:**
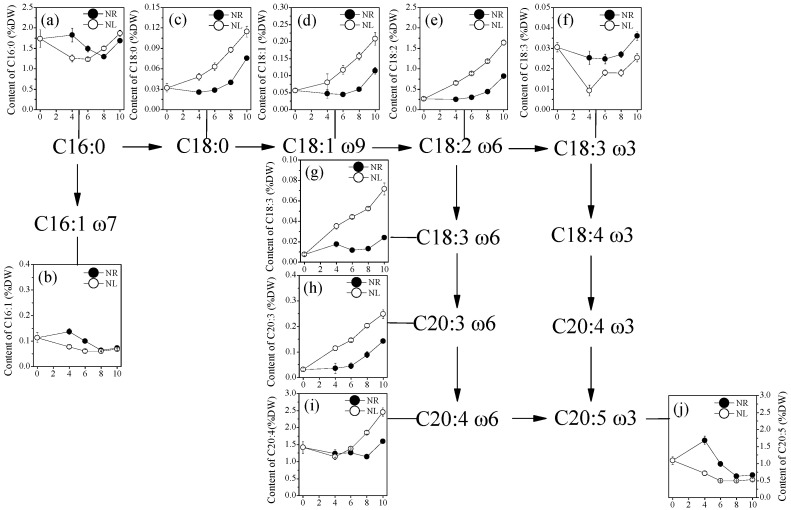
Change in fatty acids content of *Porphyridium cruentum* CCALA 415 as a function of time: (**a**) C16:0; (**b**) C16:1; (**c**) C18:0; (**d**) C18:1; (**e**) C18:2ω6; (**f**) C18:3ω3; (**g**) C18:3ω6; (**h**) C20:3ω6; (**i**) C20:4ω6; and (**j**) C20:5ω3 under N-limited and N-replete conditions. The values shown are the averages of three biological replicates and three technical replicates ± standard deviation (*n* = 9). DW: dry weight; NR: nitrogen repletion; NL: nitrogen limitation.

Fatty acids detected in *P. cruentum* CCALA 415 included C16:0, C16:1, C18:0, C18:1ω9, C18:2ω6, C18:3ω3, C18:3ω6, C20:3ω6, ARA and EPA (Figure 3). C18:4ω3 and C20:3ω6 were not detected. Of these, the fatty acids with contents exceeding 1.50% DW included ARA, C16:0, EPA, and C18:2ω6. The maximum contents of ARA (2.46% DW), C16:0 (1.87% DW), and C18:2ω6 (1.64% DW) were detected in the N-limited group on day 10, whereas that of EPA (1.68% DW) was detected in the N-replete group on day 4. It is obvious that the maximum content of EPA in the N-replete group was obtained during the exponential growth phase, while that of other fatty acids in the N-limited group was achieved at the end of the culture. Additionally, the N-limitation caused the increase in the contents of ω6 fatty acids including C18:2ω6, C18:3ω6, C20:3ω6, and ARA. At the end of the culture, the contents of C18:2ω6, C18:3ω6, C20:3ω6, and ARA in the N-limited group increased by 197.2% (*p* < 0.05), 73.8% (*p* < 0.05) and 53.4% (*p* < 0.05), respectively, compared to those in the N-replete group. In contrast, the N-limitation caused the content of ω3 fatty acids, including C18:3ω3 and EPA, to significantly decrease.

### 2.4. Profiles of Glycerolipids in P. cruentum CCALA 415

A total of 217 glycerolipid molecular species were identified in *P*. *cruentum* CCALA 415, which included 29 MGDG, 22 DGDG, 15 sulfoquinovosyldiacylglycerol (SQDG), 8 phosphatidic acid (PA), 27 PC, 14 phosphatidylethanolamine (PE), 16 phosphatidylglycerol (PG), 4 phosphatidylinositol (PI), 3 phosphatidylserine (PS), 4 monoglyceride (MAG), 21 DAG, 53 TAG and 1 free fatty acid (FA).

**Figure 4:**
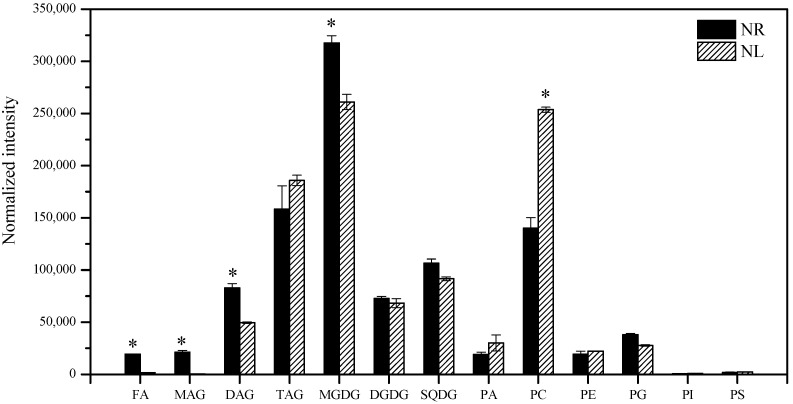
Profiles of free fatty acids and glycerolipids in *Porphyridium cruentum* CCALA 415. The symbol (*) denotes significant differences among the abundance of glycerolipids under N-limited and N-replete conditions. FA: free fatty acid; MAG: monoglyceride; DAG: diglyceride; TAG: triacylglycerol; MGDG: monogalactosyldiaclyglycerol; DGDG: digalactosyldiaclyglycerol; SQDG: sulfoquinovosyldiacylglycerol; PA: phosphatidic acid; PC: phosphatidylcholine; PE: phosphatidylethanolamine; PG: phosphatidylglycerol; PI: phosphatidylinositol; PS: phosphatidylserine.

By summing the normalized intensities of glyceride molecule species, the abundance of different glyceride molecules can be investigated (Figure 4). The most abundant glycerolipids were MGDG, PC, and TAG. For the membrane lipids, the content of prokaryotic membrane lipids (MGDG, SQDG, and PG) increased, while that of eukaryotic membrane lipids (PC and PE) decreased in response to N-limitation. For storage lipids, the N-limitation caused the content of TAG to increase, while the N-limitation caused the content of FA, MAG, and DAG to decrease (Figure 4).

**Figure 5:**
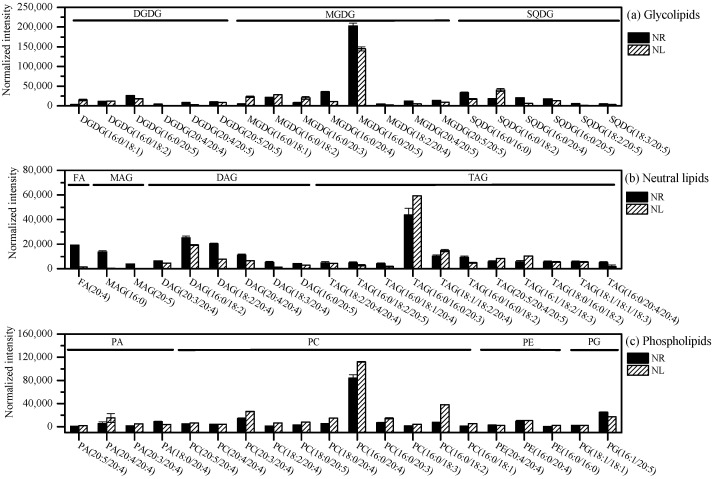
The abundance of glycolipids (**a**), neutral lipids (**b**), and phospholipids (**c**) under N-limited and N-replete conditions. FA: free fatty acid; MAG: monoglyceride; DAG: diglyceride; TAG: triacylglycerol; MGDG: monogalactosyldiaclyglycerol; DGDG: digalactosyldiaclyglycerol; SQDG: sulfoquinovosyldiacylglycerol; PA: phosphatidic acid; PC: phosphatidylcholine; PE: phosphatidylethanolamine; PG: phosphatidylglycerol.

The top 20 high-proportion glycolipids, neutral lipids, and phospholipids are listed in Figure 5. The glycerolipid species with the highest abundance were MGDG(C16:0/C20:5), DGDG(C16:0/C20:5), SQDG(C16:0/18:2), TAG(C16:0/C16:0/C20:3), MAG(C16:0), DAG(C16:0/C18:2), PC(C16:0/C20:4), PE(C16:0/C20:4), PG(C16:1/C20:5), and FA(C20:4). ARA and EPA were also detected in prokaryotic membrane lipids, such as MGDG, DGDG, SQDG, and PG. The eukaryotic membrane lipids with prokaryotic carbon skeletons were detected in PE(C16:0/C16:0) and PC(C16:0/C16:0). In addition, the prokaryotic membrane lipids with eukaryotic carbon skeletons were detected in PG(C18:1/C18:1), MGDG(C18:2/C20:4), and MGDG(C20:5/C20:5). The presence of MGDG(C16:0/C20:5) and MGDG(C20:5/C20:5) indicated that the DAG that synthesizes them might come from the inside and outside of the chloroplast, respectively. Under the N-limited condition, the abundance of MGDG(C16:0/C20:5) and DGDG(C16:0/C20:5) decreased, while that of TAG(C16:0/C16:0/C20:3) and PC(C16:0/C20:4) increased.

### 2.5. Proportion of Glycerolipid Species Containing ARA and EPA

**Figure 6:**
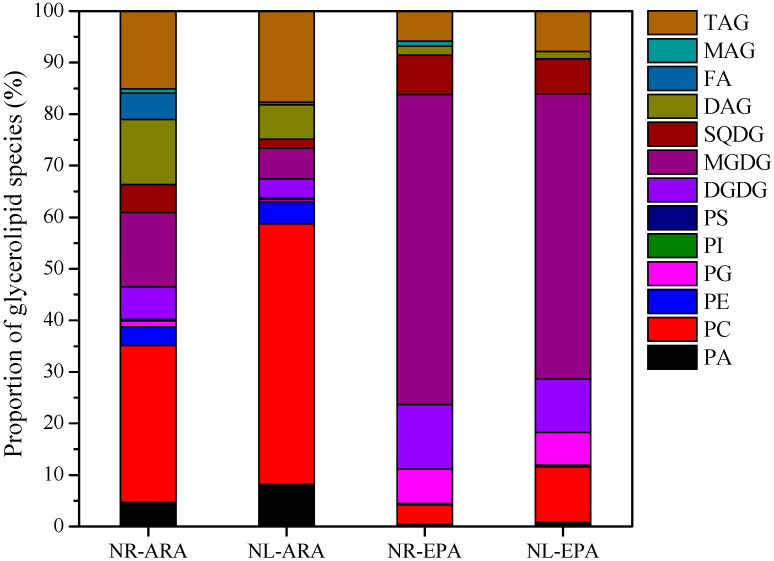
Proportion of glycerolipid species containing ARA and EPA under N-limited and N-replete conditions. DAG: diacyl glycerol; TAG: triacylglycerol; PE: phosphatidylethanolamine; PS: phosphatidylserines; PG: phosphatidylglycerol; PC: phosphatidylcholine.

As shown in Figure 6, ARA was detected in MGDG, DGDG, SQDG, PC, PE, PG, PI, PS, MAG, DAG, and TAG. It was most abundant in PC followed by TAG. In response to N-limitation, the proportion of glycerolipid species containing ARA increased from 30.4% to 50.6% for PC and from 15.1% to 17.7% for TAG. However, the proportion of glycerolipid species containing ARA decreased from 14.4% to 5.9% for MGDG and from 6.4% to 3.7% for DGDG.

Similarly, EPA was detected in MGDG, DGDG, SQDG, PC, PE, PG, PI, PS, MAG, DAG, and TAG. It was most abundant in MGDG, followed by DGDG and SQDG. Under N-limited conditions, the proportion of glycerolipid species containing EPA decreased from 60.1% to 55.4% for MGDG, from 12.6% to 10.3% for DGDG, and from 7.6% to 6.8% for SQDG. In contrast, the proportion of glycerolipid species containing EPA increased from 5.8% to 7.8% for PC.

### 2.6. Transcriptome Analysis

The G+C contents were 58.58% and 57.77% for the N-replete group and N-limited group, respectively (Supplementary Table S1). By comparing the clean data with the reference genome of *Porphyridium purpureum* in the NCBI database (GCA_008690995.1), total mapped rates for the N-replete group and N-limited group were calculated: 93.44% and 92.20%, respectively (Supplementary Table S1). A total of 9898 unigenes were successfully annotated using the NR, Swiss-Prot, Pfam, GO, KEGG, and COG databases (Supplementary Table S2). The differentially expressed genes from CCALA 415 were identified by comparing the acquired information to available TPM data. In total, 1000 genes in the N-limited condition were up-regulated and 1127 genes were down-regulated compared to the N-replete condition (Supplementary Figure S1).

**Figure 7:**
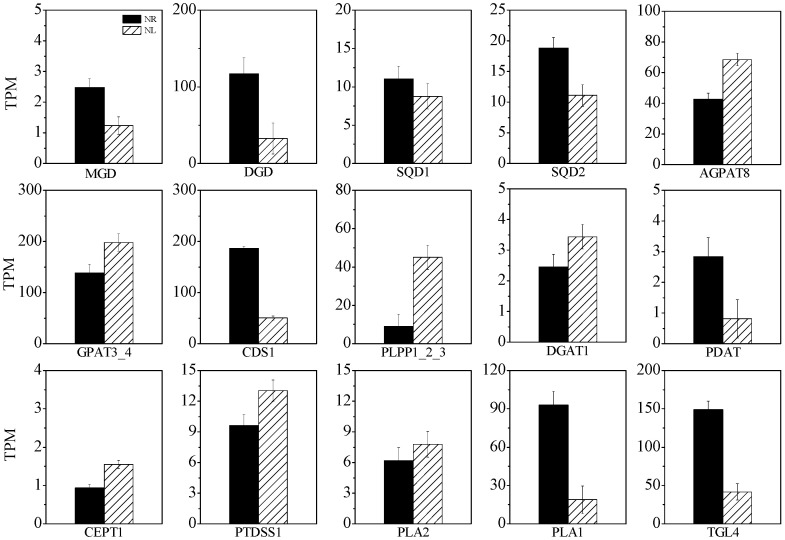
Expression dynamics of the genes involved in glycerolipid biosynthesis under N-limited and N-replete conditions. MGD: monogalactosyldiacylglycerol synthase; DGD: digalactosyldiacylglycerol synthase; SQD1: UDP-sulfoquinovose synthase; SQD2: sulfoquinovosyl transferase; GPAT: glycerol-3-phosphate acyltransferase; AGPAT: 1-acyl-sn-glycerol-3-phosphate acyltransferase; PLPP: phospholipid phosphatase; DGAT: diacylglycerol acyltransferase; CEPT1: choline/ethanolaminephosphotransferase; PTDSS: CDP-diacylglycerol-serine O-phosphatidyltransferase; CDS1: phosphatidate cytidylyltransferase; PDAT: phospholipid diacylglycerol acyltransferase; PLA1: phospholipase A1; TGL4: triacylglycerol lipase; PLA2: phospholipase A2.

As shown in Figure 7, monogalactosyldiacylglycerol synthase (MGD) and digalactosyldiacylglycerol synthase (DGD) are key enzymes for the synthesis of MGDG and DGDG. Under the N-limited condition, the expression levels of MGD and DGD were decreased by 50.5% and 72.3%, respectively, whereas those of UDP-sulfoquinovose synthase (SQD1) and sulfoquinovosyl transferase (SQD2), enzymes involved in the synthesis of SQDG, were reduced by 20.6% and 41.0%, respectively. The expression levels of key enzymes in the Kennedy pathway involved in TAG synthesis, including glycerol-3-phosphate acyltransferase (GPAT), 1-acyl-sn-glycerol-3-phosphate acyltransferase (AGPAT), phospholipid phosphatase (PLPP), and diacylglycerol acyltransferase (DGAT), were increased by 42.6%, 60.7%, 394.0%, and 40.0%, respectively. DAG synthesized in the Kennedy pathway is an important precursor for the synthesis of PC, PE, and PS. Choline/ethanolaminephosphotransferase (CEPT1) is involved in the conversion of DAG to PC and PE, and CDP-diacylglycerol-serine O-phosphatidyltransferase (PTDSS) is involved in the synthesis of PS from PC. Under N-limited conditions, the expression levels of CEPT1 and PTDSS were up-regulated by 65.8% and 35.2%, respectively. The expression level of the phosphatidate cytidylyltransferase (CDS1) gene, which encodes an enzyme participating in the conversion of PA to CDP-DAG, an important substance for the synthesis of PI and PG, was reduced by 73.2%. The expression of phospholipid diacylglycerol acyltransferase (PDAT), which is responsible for the conversion of phospholipids to TAG, was down-regulated by 71.5%. The expression of gene-encoding phospholipase A1 (PLA1), which is involved in the hydrolysis of glycolipids to fatty acyl-CoA, was down-regulated by 79.7%. The expression level of the triacylglycerol lipase (TGL4) gene, which encodes an enzyme responsible for hydrolyzing glycerolipids to fatty acyl-CoA, was reduced by 72.2% under N-limited conditions. The expression level of gene-encoding phospholipase A2 (PLA2), an important enzyme that hydrolyzes phospholipids to fatty acyl-CoA, was increased by 26.1%.

**Figure 8:**
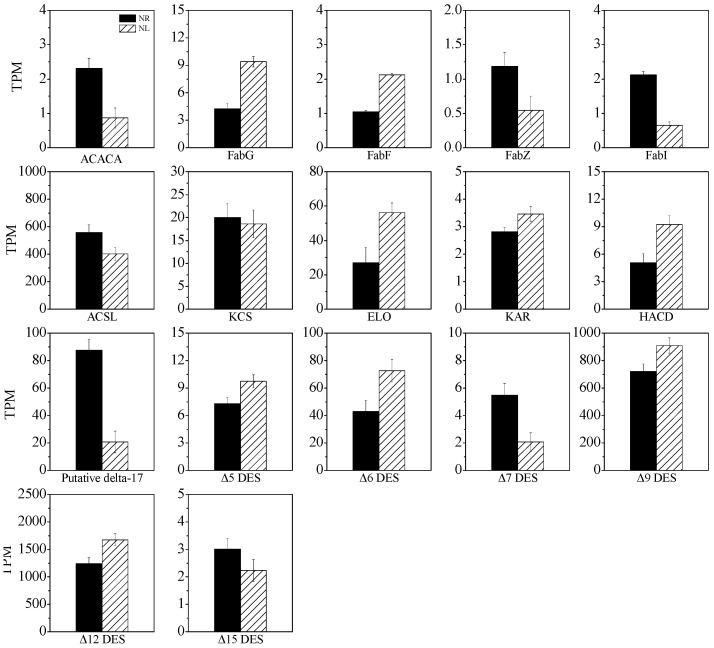
Expression dynamics of the genes involved in fatty acid biosynthesis under N-limited and N-replete conditions. Acetyl-CoA carboxylase (ACACA); fatty acyl-CoA synthetase (ACSL); 3-ketoacyl-CoA synthase (KCS); elongation of fatty acid protein (ELO); very-long-chain 3-oxoacyl-CoA reductase (KAR); very-long-chain (3R)-3-hydroxyacyl-CoA dehydratase (HACD).

As shown in Figure 8, the expression level of gene-encoding acetyl-CoA carboxylase (ACACA), a key enzyme in de novo fatty acid synthesis responsible for catalyzing acetyl-CoA to malonyl-CoA, was down-regulated by 62.6% under N-limited conditions. There are four key genes involved in de novo fatty acid synthesis, i.e., FabF, FabZ, FabI, and FabG. The expression levels of FabZ and FabI were reduced by 54.0% and 69.1%, respectively, while those of FabF and FabG were increased by 101% and 121%, respectively. Under N-limited conditions, the level of fatty acyl-CoA synthetase (ACSL), an enzyme catalyzing fatty acid ACP to fatty acid CoA, was decreased by 28.2%. Additionally, under N-limited conditions, the expression levels of the Δ9FAD gene, which encodes an enzyme catalyzing C18:0 to C18:1, and the Δ12FAD gene, which encodes an enzyme catalyzing C18:1 to C18:2ω6, were increased by 26.3% and 34.8%, respectively. Δ15FAD, which is the first key enzyme in the ω3 fatty acid pathway, catalyzes the synthesis of C18:3ω3 from C18:2ω6. The expression of the Δ15FAD gene was down-regulated by 25.7%. The gene expression levels of Δ5FAD and Δ6FAD were increased by 33.3% and 68.9%, respectively. The expression level of genes encoding long-chain fatty acid chain elongases, including 3-ketoacyl-CoA synthase (KCS), elongation of fatty acid protein (ELO), very-long-chain 3-oxoacyl-CoA reductase (KAR), and very-long-chain (3R)-3-hydroxyacyl-CoA dehydratase (HACD), were up-regulated to varying degrees. In addition, a special gene that catalyzes the desaturation of C20 fatty acids had a particularly high expression level. The expression level of this gene under N-replete conditions was 3.21 times higher than that under N-limited conditions (*p* < 0.05).

## 3. Discussion

Nitrogen, one of the essential elements for microalgae growth, participates in the synthesis of photosynthetic pigments, key enzymes, proteins, DNA, ATP, and membrane lipids [22]. In the present study, nitrogen limitation significantly inhibited cell division, which is consistent with the results reported in Huerlimann et al. (2014) [23]. Compared with that in the N-replete group (nitrate concentration = 1.00 g L^−1^), *P. purpureum* CoE1 in the N-limited group (nitrate concentration = 0.25 g L^−1^) had a slower growth rate and a more quickly ended exponential growth phase [24]. In addition, the change in color of the culture was mainly caused by the degradation of nitrogen-containing phycoerythrin (Figure 1c). A large number of polysaccharide particles were observed in the algal cells, with only a small amount of oil bodies (Figure 1d), which indicates that the main storage carbon in *P*. *cruentum* CCALA 415 was in the form of intracellular polysaccharide rather than triacylglycerol. This finding is consistent with a previous study, in which Floridean starch was found to be used as a storage carbon by most red algae [25].

Nitrogen limitation can promote the accumulation of storage lipids in oleaginous microalgae, such as *Nannochloropsis* [26], *Chlorella* [27], and *Eustigmatos* [12]. Microalgae with a total lipid content of more than 30% DW are defined as oleaginous microalgae. In the present study, the maximum total lipid content in *P*. *cruentum* CCALA 415 was only 11.01% DW, and the membrane lipids accounted for almost 50% of the total lipids (Figure 2); thus, *P*. *cruentum* CCALA 415 cannot be classified as an oleaginous microalga. Under N-limited conditions, the storage lipid content in *P*. *cruentum* CCALA 415 significantly increased, whereas the membrane lipid content significantly decreased (Figure 2). Similar trends have also been observed in *N. oceanica* [21] and *Eustigmatos vischeri* [12]. The content of membrane lipids in *E*. *vischeri* JHsu-01 grown in N-limited conditions (3.5 mmol L^−1^ NaNO_3_) significantly decreased from 9.2% to 4.5% DW. In contrast, the content of storage lipids in *E. vischeri* JHsu-01 grown in N-limited conditions (3.5 mmol L^−1^ NaNO_3_) reached 47.9% DW, which was 12.1% higher than that of the N-replete group (35.8% DW) [12]. The TAG content in *N. oceanica* increased from 0.4% to 31.1% DW after 7 days of culture. However, the membrane lipid content decreased by 30% [21]. In our study, the increase in the number of intracellular oil bodies and the destruction of the thylakoid structure also proved that N-limitation can enhance the accumulation of storage lipids and the decomposition of membrane lipids (Figure 1b,d).

ARA, C16:0, EPA, and C18:2ω6 were found to be the main fatty acids in *P. cruentum* CCALA 415, which is consistent with the findings in most previous reports [3,28,29,30,31]. However, there were some differences in other fatty acids with low contents. Shiran et al. found C16:3, C20:2ω6, and C20:4ω3 in *P. purpureum* [29]. Cohen et al. detected C18:4ω3 and C20:3ω3 in *P. cruentum* [30]. Huang et al. identified C14:0, C20:1, and C20:2ω6, but not C18:3ω6, in *P. purpureum* [31]. These differences might be related to *Porphyridium* species, culture conditions, and fatty acid determination methods. In the present study, the contents of ARA and EPA were 1.14–2.46% DW and 0.49–1.67% DW, respectively (Figure 3). Huang et al. reported that the contents of ARA and EPA in *P. purpureum* were 1.18% DW and 1.84% DW, respectively [31], whereas Jiao et al. reported that the contents were 1.5% DW and 0.7% DW, respectively [32]. In *P. cruentum* CCALA 415, all the fatty acids in the ω6 fatty acid pathway were detected, but two fatty acids (C18:4ω3 and C20:4ω3) in the ω3 fatty acid pathway were not detected (Figure 3). Other than the studies by Shiran et al. and Khozin et al., there are no other reports on the presence of the above two fatty acids in *Porphyridium* [3,29]. These two fatty acids have also not been detected in EPA-producing microalgae, such as *N. oceanica* [18], *P. tricornutum* [33] and *Parietochloris incise* [17]. There are two possible reasons for this result: (1) the microalgae may not synthesize C18:4ω3 and C20:4ω3; and (2) their contents are lower than the detectable levels. Based on the current results, we cannot draw a conclusion. In the following sections, we make reasonable assumptions in conjunction with transcriptome data.

N-limitation promoted the synthesis of ω6 fatty acids (C18:2ω6, C18:3ω6, C20:3ω6, and ARA), while it inhibited the synthesis of ω3 fatty acids (C18:3ω3 and EPA) (Figure 3). Similar results have been reported in previous studies. The percentages of EPA in the total fatty acids in *P. purpureum* SCS-02 were 16.36% in 3.5 mmol L^−1^ KNO_3_ and 28.38% in 17.6 mmol L^−1^ KNO_3_ [34]. The contents of EPA in *N. oceanica* IMET1 were 2.62% DW in 2.20 mmol L^−1^ KNO_3_ and 1.77% DW in 0.06 mmol L^−1^ KNO_3_ [35]. Other observations have also provided different results. N-limitation caused the ARA and EPA contents in *P. purpureum* CoE1 to significantly increase [24]. Nitrogen depletion had no effect on the ARA content in *N. oculata* but caused a reduction in the EPA content from 1.9% to 1.6% DW [36]. It has been speculated that the inhibition of EPA production might be related to Δ15FAD. Δ15FAD is the first key rate-limiting enzyme in the ω3 fatty acid synthesis pathway, mainly localized in the chloroplast membrane; its function is to catalyze the conversion of C18:2ω6 to C18:3ω3 [37]. Under N-limited conditions, the degradation of the chloroplast membrane led to a decrease in Δ15FAD activity, thereby inhibiting EPA synthesis. However, the synthesis of ω6 fatty acids requires the participation of Δ6FAD and Δ5FAD, both of which are localized in the ER membrane instead of the chloroplast membrane.

The lipidomics results show that prokaryotic membrane lipid MGDG had the highest abundance, followed by eukaryotic membrane lipid PC and storage lipid TAG (Figure 4). The contents of PC and TAG increased, while that of MGDG decreased, under N-limited conditions (Figure 4). This is possibly due to the fact that MGDG is synthesized in the chloroplast, whereas PC and TAG are synthesized in the ER [8]. Under N-limited conditions, the high degree of degradation of chloroplasts resulted in the inhibition of MGDG synthesis, while this had a minor effect on PC and TAG synthesis. Similar results have been reported in oleaginous microalgae. Under nitrogen deficiency conditions, the TAG content in *N. oceanica* IMET1 significantly increases, while the MGDG content decreases [15]. In our study, EPA was mainly distributed in MGDG (C16:0/C20:5), while ARA was mainly distributed in PC (C16:0/C20:4) (Figure 6). Carter et al. and Li et al. have shown that the lipid with a high content in *P. purpureum* is DGDG (C20:5/C16:0) [38,39], while Shiran et al. and Khozin et al. have described that the high-content lipid in *P. cruentum* is MGDG [3,29]. It is likely that different algal species, culture conditions, and lipidome databases led to these different results. EPA and ARA should be synthesized in the ER, where Δ5FAD and Δ6FAD are localized. Theoretically, there should be more PC and TAG containing EPA, and DAG containing ARA should also have the same opportunity to be shuttled into the chloroplast. In addition, there should be a large amount of MGDG molecules containing ARA. However, the findings show that there was almost no EPA in PC and ARA in MGDG (Figure 6). Thus, transcriptomics was employed to further analyze these findings to find possible reasons behind them.

The expression of genes involved in the de novo fatty acid synthesis pathway, including ACACA, FabZ, FabI, and ACSL [33], were down-regulated to varying degrees under N-limited conditions, indicating that the de novo fatty acid synthesis was weakened. The expression of several genes involved in the de novo fatty acid synthesis pathway is down-regulated in *P. purpureum* grown under nitrogen deficiency [26]. It could be speculated that the degradation of the chloroplast might greatly inhibit fatty acid synthesis by this pathway, which occurs in the chloroplast. The expressions of the ELOVL, Δ6FAD, and Δ5FAD genes were up-regulated under N-limited conditions, which is consistent with the findings reported by Ji et al. [40]. However, different observations have also been reported. The expression of Δ5FAD was up-regulated and was not significantly different from that of Δ6FAD in *Isochrysis* aff. *galbana* cultured under nitrogen-deficient conditions [23]. Han et al. identified a total of seven genes encoding ELOVL in *N. oceanica* IMET1, and observed that the expressions of these genes are down-regulated under N-deprivation conditions [15]. Two Δ5FAD gene variants were identified in IMET1. The expression level of one variant was low under N-replete conditions but was significantly increased 2-fold under N-limited conditions. Conversely, the other variant had high expression levels under both N-replete and N-limited conditions. Thus, it could be concluded that the two Δ5FAD variants were involved in the synthesis of EPA under N-limited and N-replete conditions [15].

Δ9FAD is a soluble enzyme localized in the cytoplasm of chloroplasts that mainly catalyzes the conversion of C18:0 to C18:1ω9 [8]. In the present study, the expression level of the Δ9FAD gene was significantly enhanced under N-limited conditions. Ji et al. reported that the expression of Δ9FAD in *P. purpureum* is up-regulated, which is consistent with our study [40]. Δ12FAD is the most abundant desaturase that catalyzes the conversion of C18:1ω9 to C18:2ω6; it is located in both the chloroplast and the ER [8]. The present study showed that N-limitation enhanced the expression level of Δ12FAD, and this may explain why the C18:2ω6 content was increased under N-limited conditions. Ji et al. showed that the expression level of Δ12FAD was also up-regulated in *P*. *purpureum* cultured under nitrogen-deficient conditions [40]. Δ15FAD is a typical membrane protein found in the chloroplast membrane and ER membrane [8]. The substrates of Δ15FAD are PC in the ER and all types of glycerides in the chloroplast. In addition, Δ15FAD is the first and the rate-limiting enzyme in the ω3 fatty acid synthesis pathway [37]. The expression level of Δ15FAD in *P. cruentum* CCALA 415 was the lowest among all desaturases, and this might be the main reason for the low content of C18:3ω3. The expression levels of Δ5FAD and Δ6FAD in *P. cruentum* CCALA 415 were high. The intermediary metabolic fatty acids C18:4ω3 and C20:4ω3 were not detected. It is speculated that the low substrate concentration of C18:3ω3 (substrate) and high enzymatic activities of Δ5FAD and Δ6FAD might lead to the high conversion of C18:4ω3 and C20:4ω3 within a very short period of time, causing them to become undetectable in the study. Ji et al. reported that C18:4ω3 in the ω3 pathway was not detected, and they believed that the ω3 pathway might not be present or might play a minor role in *P. purpureum* [40]. Taken together, we speculate that the synthesis of ARA and EPA in *P. cruentum* CCALA 415 was mainly achieved through the ω6 fatty acid synthesis pathway.

The expressions of GPAT, AGPAT, PLPP, and DGAT, which are genes encoding enzymes involved in the Kennedy synthesis pathway of TAG, were up-regulated under N-limited conditions, indicating that TAG synthesis was enhanced [8]. DGAT has strong substrate specificity and is the key rate-limiting enzyme for TAG synthesis [18]. Xin et al. reported two new PUFA-preferring enzymes in *N. oceanica* that could distinguish between different PUFAs during TAG synthesis, i.e., NoDGAT2J for linoleic acid and NoDGAT2K for EPA. There are two isoforms of DGAT, i.e., DGAT1 and DGAT2; they have no apparent sequence similarity but catalyze the same reaction [18]. Liu et al. obtained DGAT from the ARA-rich green alga *Myrmecia incisais* [41]. Compared with other DGAT members, DGAT2A (rather than DGAT2C) is the main contributor to the incorporation of ARA into TAG [41]. The expression of TGL4, a gene encoding triacylglycerol lipase, was significantly down-regulated under N-limited conditions, indicating that the degradation of TAG was attenuated. PLA1 is a gene encoding galactolipase that catalyzes the degradation of MGDG and DGDG to fatty acyl-CoA [42]. The generated fatty acyl-CoA can cross the chloroplast membrane into the cytoplasm to participate in the synthesis of eukaryotic membrane lipids. The expression of PLA1 was up-regulated under N-limited conditions; thus, the catabolism of glycolipids was enhanced, in turn causing the transport of fatty acyl-CoA to the chloroplast to be enhanced. EPA synthesized in the chloroplast by Δ17FAD might be exported to the ER through this pathway. This might be one of the reasons why EPA in PC was increased under N-limited conditions. PLA2G is a gene that encodes phospholipase, an enzyme that breaks down phospholipids to fatty acyl-CoA, which is the main pathway for C18:3ω6 synthesis. C20:3ω6 is generated from C18:3ω6 by the action of ELOVL and is then used by Δ5FAD to form ARA. The up-regulation of PLA2G, Δ5FAD, and Δ6FAD expression levels also indicated that ARA synthesis was enhanced. The genes for synthesizing MGDG, DGDG, and SQDG were down-regulated, while the genes for synthesizing TAG, PC, PE, and PS were up-regulated. This might be the result of a series of cellular responses caused by chloroplast degradation, as the synthesis of MGDG, DGDG, and SQDG occurred in chloroplasts, while the synthesis of TAG, PC, and PE occurred in the endoplasmic reticulum.

**Figure 9:**
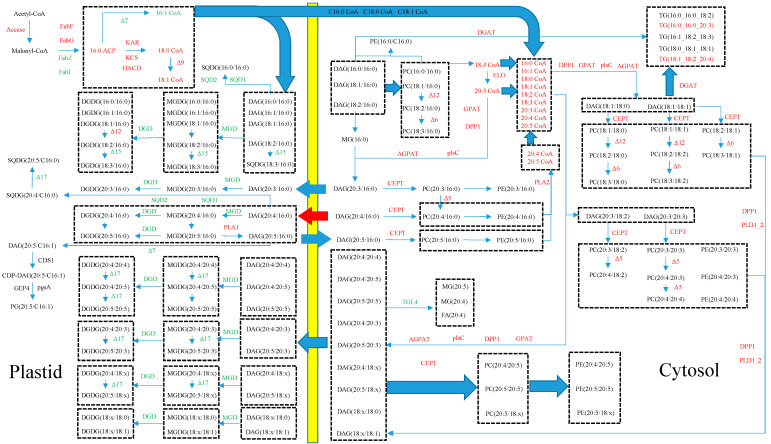
Conceptual EPA biosynthesis network in *Porphyridium cruentum* CCALA 415.

It is speculated that the distribution of Δ17FAD in different cellular compartments and the low activity of Δ15FAD played a key role in the distribution of EPA in MGDG. The expression level of Δ15FAD was very low, implying that the majority of EPA synthesized in *P. cruentum* CCALA 415 was not created through the ω3 pathway. A large amount of EPA was more likely derived from the ω6 pathway through the desaturation of ARA by Δ17FAD. We mapped the hypothetical metabolic network of EPA synthesis in *P. cruentum* CCALA 415 based on the obtained transcriptomic and lipidomic data in Figure 9. If the ω6 pathway of EPA in *P. cruentum* CCALA 415 exists, the following two questions will need to be addressed: (1) how does extracellular ARA enter the chloroplast? and (2) does Δ17FAD exist in the chloroplast? To address the first question, we found that the abundance of two types of DAG, i.e., DAG(C20:4/C18:2) and DAG(C20:4/C16:0), both of which are important cross-talking molecules between the chloroplast and the ER, were very high. They might act as carriers to transport ARA synthesized in the ER into the chloroplast. DAG(C20:4/C18:2) that is shuttled into the chloroplast is first decomposed into MAG(C20:4), which is then converted into DAG(C20:4/C16:0). MGDG(C20:4/C16:0) is synthesized from DAG(C20:4/C16:0) by MGD, and MGDG(C20:5/C16:0) is synthesized by Δ17FAD. In respect of the second question, there were two pieces of evidence indicating that Δ17FAD may exist in the chloroplast. A large amount of PC(C20:4/C16:0), along with a small amount of PC(C20:5/C16:0), indicated that Δ17FAD was not present in the ER. Similarly, a large amount of MGDG (C20:5/C16:0) and a small amount of MGDG(C20:4/C16:0) indicated that Δ17FAD was present in the chloroplast. MGDG(C20:4/C16:0) might be rapidly converted to MGDG(C20:5/C16:0) by Δ17FAD, causing the abundance of MGDG(C20:4/C16:0) to be low. Δ17FAD has been identified in many fungi species; it can use the acyl-CoA in phospholipids as a substrate. Three ω3 desaturases from *Pythium aphanidermatum*, *Phytophthora sojae*, and *Phytophthora ramorum* are only 55% sequence-homologous to the known Δ17FAD in *Saprolegnia diclina* [16]. Until recently, only Shiran et al. and Khozin et al. had reported the possible presence of Δ17FAD in *P. cruentum* [3,29]; however, detailed information about the enzyme was not provided in the report. According to our results, we may conclude that Δ17FAD is present in the chloroplasts and can use glycolipids as substrates.

## 4. Materials and Methods

### 4.1. Algae Species and Cultivation Methods

*P. cruentum* CCALA 415 was purchased from the Culture Collection of Autotrophic Organisms in the Czech Republic. *P. cruentum* CCALA 415 was cultured in a Ø3.0 cm × 60 cm glass column photobioreactor with ASW medium. Illumination was provided by T8 fluorescent lamps (Philips; Suzhou, China) at 200 μmol photons m^−2^ s^−1^. The culture temperature was maintained at 25 ± 1 °C. CO_2_-enriched compressed air (1% CO_2_ in volume) was continuously bubbled into the photobioreactor to provide a carbon source. To minimize bacterial and fungal contamination, all culture vessels and media were autoclaved, and CO_2_-enriched compressed air was bubbled into the photobioreactors using a sterile filter (0.22 μm).

### 4.2. Experimental Design

*P. cruentum* CCALA 415 was grown in ASW medium for 7 days. The algal cells were then collected via centrifugation at 3000 rpm for 5 min and washed 1–2 times with nitrogen-free ASW medium. The concentrated algal cells were then inoculated into two nitrogen concentration treatments: (1) 1.5 g L^−1^ KNO_3_ (N-replete group); (2) 0.3 g L^−1^ KNO_3_ (N-limited group). The initial optical density at the wavelength of 750 nm was 0.50 ± 0.02. Three biological replicates were set for each treatment. On days 0, 2, 4, 6, 8, and 10, the cell count was determined and cell morphology was observed; the freeze-dried biomass was used to determine lipid fractions and fatty acid content. On day 8, the algal cells were collected for transcriptomic and lipidomic analyses.

### 4.3. Growth Measurement and Morphological Observation

Cell counts were measured from day 0 to day 10 using an XB-K-25 hematocytometer (Shanghai Qijing Biochemical Reagent Instrument; Shanghai, China). Each measurement was performed in triplicate. Optical morphological examination was performed from day 0 to day 10 under a BX41 light microscope (Olympus; Tokyo, Japan). Cellular ultrastructure observation was carried out on day 8. The method for specimen preparation for transmission electronic microscopy was as described by Li et al. [27].

### 4.4. Determination of Lipid Content

The freeze-dried biomass (100 mg) was used to determinate the contents of membrane lipids (glycolipids and phospholipids) and storage lipids (neutral lipids). A modified Khozin–Goldberg method was employed to extract total lipids [43]. Approximately 20 mg of the extracted total lipids were separated into membrane lipids and neutral lipids using 500 mg of a Cleanert silica gel column (Agela Technologies; Tianjin, China) [12]. Ten milliliters of chloroform and methanol were used to elute storage lipids and membrane lipids, respectively. Each lipid fraction was dried under a gentle nitrogen stream and weighed. Each measurement was performed in triplicate.

### 4.5. Determination of Fatty Acid Compositions and Contents

The fatty acid compositions and contents were determined using the method previously described by Xu et al. [12]. The freeze-dried biomass (25 mg) was trans-methylated with 2% H_2_SO_4_ in a methanol:toluene mixture (90:10, *v*:*v*) at 80 °C for 1.5 h. The contents of fatty acid methyl esters (FAMEs) were determined using a GC-2014 gas chromatograph spectrometer with a flame ionization detector (Shimadzu; Kyoto, Japan), equipped with a 30 m fused silica DB-WAX capillary column (Agilent Technologies; Santa Clara, CA, USA). The temperature of the injection port was maintained at 260 °C. The column temperature was programmed from 140 °C to 240 °C at 10 °C min^−1^ with a hold of 5 min at 240 °C. High-purity argon was used as the carrier gas at a flow rate of 1.2 mL min^−1^. Individual peaks of FAMEs were identified through comparisons of retention times with 37 fatty acid standards (Nu-Chek-Prep; Elysian, MN, USA). Each measurement was performed in triplicate.

### 4.6. Lipidomics Analyses

Approximately 25 mg of freeze-dried biomass was used to extract the crude lipid by adding 750 μL chloroform/methanol solution (2:1, −20 °C) with 100 mg glass beads to a 2 mL centrifuge tube. The centrifuge tube was immersed in liquid nitrogen for freezing and then was taken out at room temperature for thawing. The centrifuge tube was installed in a SCIENTZ-48 bead homogenizer (Huakeda; Wuhan, China) for 2 min at 70 Hz. The above steps were repeated twice. An amount of 0.19 mL ddH_2_O was added to the centrifuge tube and vortexed for 30 s. The extract was centrifuged at 12,000 rpm for 5 min at room temperature and 300 μL lower-layer fluid was transferred to a new centrifuge tube. A total of 500 μL of chloroform/methanol solution (2:1, −20 °C) was added to the new centrifuge tube and vortexed for 30 s. The extract was then centrifuged at 12,000 rpm for 5 min at room temperature, and 400 μL lower-layer fluid was transferred to the same centrifuge tube as above. The crude lipid extract was concentrated in a vacuum and dissolved with 200 μL isopropanol. The separation of lipid fractions was conducted on an Ultimate 3000 high-performance liquid chromatography (HPLC) system (Thermo Fisher, Dreieich, Germany) equipped with an ACQUITY UPLC^®^ BEH C18 column (100 mm × 2.1 mm, 1.7 µm). The gradient elution procedure of HPLC for the separation of lipid fractions and the mass spectrometry parameters were as described by Li et al. [39]. The lipidomic analyses were performed in duplicate for each nitrogen condition.

### 4.7. Transcriptome Analyses

Microalgae cells were collected on day 8 (mid-logarithmic growth phase) for transcriptome determination under N-limited and N-replete conditions via centrifugation (8000× *g* at 25 °C for 5 min) and stored at −80 °C. RNA extraction, cDNA library construction, and sequencing were carried out according to the method described by Xu et al. [12]. The data were arranged by removing linker sequences and low-quality reads from raw data and assembled using short-read assembly software (Trinity Version v2.8.5). All unigenes were annotated using BLAST with a cutoff E-value of 10^−5^. The data were then compared to NR, NT, Swiss-Prot, KEGG, COG, and GO databases to analyze the most descriptive annotation of each sequence. The transcripts per million (TPM) method was applied to calculate the expression of unigenes. A false discovery rate of <0.001 and log_2_ (fold change) of ≥±1 were set as the thresholds to determine the significance of gene expression differences.

### 4.8. Statistical Analysis

Student’s *t*-test was used to compare the cell counts, lipid contents, and fatty acid contents between two data points (N-replete and N-limited conditions, *n* = 9). If Student’s *t*-test resulted in *p* < 0.05, the difference was interpreted as being significant. For lipidomic analyses, a multivariate analysis was performed to compare the two groups using principal component analysis (PCA), partial least-squares discriminant analysis (PLS-DA), and orthogonal partial least-squares discriminant analysis (OPLS-DA).

## 5. Conclusions

MGDG (C16:0/C20:5) and PC (C16:0/C20:4) were found to be the main glycerolipid molecules in *P. cruentum* CCALA 415, while ARA was mainly distributed in PC, and EPA was mainly distributed in MGDG. Additionally, N-limitation has an impact on the distribution of ARA and EPA in different glycerolipid molecules. Only a small proportion of EPA was synthesized through the ω3 pathway, while the majority of EPA was synthesized through the ω6 pathway. This observation suggests that ARA synthesized in the ER was likely shuttled into the chloroplast by DAG and was converted into EPA by Δ17FAD.

## Data Availability

The data presented in this study are available from the corresponding author upon request.

## Supplementary Materials

The following supporting information can be downloaded at: https://www.mdpi.com/article/10.3390/md22020082/s1. Figure S1: Statistics of differentially expressed genes in *Porphyridium cruentum* CCALA 415 under N-limited and N-replete conditions. Table S1: Summary of output statistics by Illumina sequencing. Table S2: The annotation statistics of unigenes in *Porphyridium cruentum* CCALA 415.
