# Supplementary material for: Reconstruction of Long-Chain Polyunsaturated Acid Synthesis Pathways in Marine Red Microalga Porphyridium cruentum Using Lipidomics and Transcriptomics
Source: Mar Drugs. 2024 Feb 9;22(2):82. doi: 10.3390/md22020082 (PMC10890038; doi:10.3390/md22020082)
Supplement: Supplementary file 1 [file marinedrugs-22-00082-s001.zip › marinedrugs-2818312-supplementary.pdf]

Supplementary Data

MANUSCRIPT TITLE: Reconstruction of long-chain polyunsaturated acids synthesis pathways in marine microalga Porphyridium cruentum using lipidomics and transcriptomics

AUTHORS: Tao Li [1], Chulin Li [1], Weinan Wang [1], Hualian Wu [1], Houbo Wu [1], Jin Xu [2*] and Wenzhou Xiang [1*]

ADDRESS: 1. CAS Key Laboratory of Tropical Marine Bio-resources and Ecology, Guangdong Key Laboratory of Marine Materia Medica, Institution of South China Sea Ecology and Environmental Engineering, South China Sea Institute of Oceanology, Chinese Academy of Sciences, Guangzhou 510301, China

2. Guangzhou Institute of Energy Conversion, Chinese Academy of Sciences, CAS Key Laboratory of Renewable Energy, Guangdong Provincial Key Laboratory of New and Renewable Energy Research and Development, Guangzhou 510640, China

NO. OF TABLES: 2

NO. OF FIGURES: 1

NO. OF PAGES: 4

Table S1. Summary of output statistics by Illumina sequencing

Sample	Raw reads	Clean reads	Error rate(%)	Q30(%)	GC content	Total reads	Total mapped	Total mapped(%)
N-limitation	49327784	48762135	0.026	93.35	58.58	48762135	44972016	92.20
N-repletion	47751462	47199666	0.026	93.52	57.77	47199666	44103622	93.44

Table S2 The annotation statistics of unigenes in *Porphyridium cruentum* CCALA 415

	GO	KEGG	COG	NR	Swiss-Prot	Pfam	Total
Unigenes	6364	3879	6136	6989	5439	7082	9898

*NR: Non-redundant protein sequence database; NT: Nucleotide Sequence Database; KEGG: Kyoto Encyclopedia of Genes and Genomes; COG: clusters of orthologous groups; GO: Gene ontology

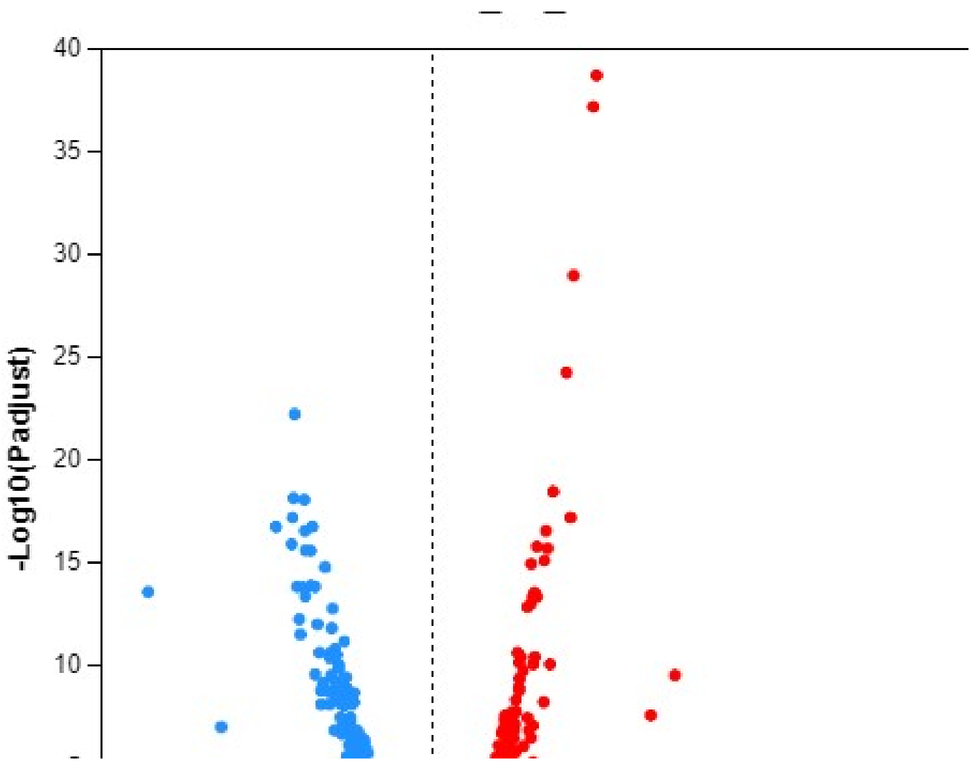

Figure S1. Statistics of differentially expressed genes in *Porphyridium cruentum* CCALA 415 under N-limited and N-replete conditions
